# That’s not funny! – But it should be: effects of humorous emotion regulation on emotional experience and memory

**DOI:** 10.3389/fpsyg.2015.01296

**Published:** 2015-08-28

**Authors:** Lisa Kugler, Christof Kuhbandner

**Affiliations:** Department of Psychology, University of RegensburgRegensburg, Germany

**Keywords:** emotion regulation, humor, reappraisal, memory, coping

## Abstract

Previous research has shown that humorous reappraisal can reduce elicited negative emotions, suggesting that humor may be a functional strategy to cope with emotionally negative situations. However, the effect of humorous reappraisal on later memory about the emotion-eliciting situation is currently unknown, although this is crucial for more adaptive responding in future situations. To address this issue, we examined the effects of humorous reappraisal on both emotional experience and memory, compared to non-humorous rational reappraisal and a non-reappraisal control condition. Replicating previous findings, humorous reappraisal reduced evoked negative valence and arousal levels very effectively, and the down-regulation of experienced negative emotions was even more pronounced after humorous compared to rational reappraisal. Regarding later memory for emotion-eliciting stimuli, both humorous and rational reappraisal reduced free recall, but recognition memory was unaffected, with memory strength being stronger after humorous than after rational reappraisal. These results indicate that humor seems to be indeed an optimal strategy to cope with negative situations because humor can help us to feel better when confronted with negative stimuli, but still allows us to retrieve stimulus information later when afforded to do so by the presence of appropriate contextual features.

## Introduction

A central question of emotion research is how to functionally regulate evoked negative emotional experiences. As suggested early in psychoanalytic theory (Freud, 1905/1960, 1928), one promising strategy to functionally regulate negative emotional experiences may be humor. Indeed, such an assumption seems to be supported by more recent experimental research, showing that viewing negative stimuli in a humorous way can reduce the strength of elicited negative emotions (e.g., Strick et al., 2009; Samson and Gross, 2012; Samson et al., 2014). Such beneficial effects of humor have been attributed to a number of mechanisms such as cognitive distraction from negative stimuli (e.g., Strick et al., 2009), cognitive reappraisal of negative stimuli in less threatening ways (e.g., Samson and Gross, 2012), and an “undoing” of negative by positive emotions (Fredrickson et al., 2000).

However, in order to more fundamentally evaluate the functionality of an emotion regulation strategy, it is not enough to look at the effects of emotion regulation on the strength of emotional responding in the current situation. Rather, it is additionally important to take into account the effects of emotion regulation on later memory about the emotion-eliciting event (e.g., Richards and Gross, 2000). Basically, emotions are assumed to exist for the sake of signaling the consequences of a stimulus for one’s motives and goals (e.g., Frijda, 1988), with negative emotions signaling that stimuli may be harmful. Accordingly, in order to prepare the organism for a more adaptive responding in future situations, it would be adaptive to retain the emotion-eliciting stimuli as well as possible, an assumption which is supported by the fact that later memory for negative stimuli is typically enhanced compared to neutral stimuli (see Hamann, 2001, for a review). Thus, if an emotion regulation strategy would down-regulate negative emotional experiences at the cost of reduced memory for the emotion-eliciting event, it may help in the short term to cope with negative emotional experiences in the current situation, but it may be detrimental for a more adaptive responding to the negative event in future situations.

Although the effects of humor on the strength of elicited negative emotions when confronted with negative stimuli have been examined in previous research, to our knowledge, research on the effects of humor on later memory about negative stimuli is lacking. In particular, the suggested mechanisms that may underlie the effects of humor on experienced emotions make rather different predictions about how humor may affect later memory. If the beneficial effect of humor on experienced emotions is based on the mechanism that humorous processing requires attentional resources so that people are distracted from negative stimuli (Strick et al., 2009), later memory for negative stimuli should be decreased because attention is a prerequisite for later memory (e.g., Mulligan, 2008), an assumption which is supported by the finding that emotion regulation by distraction seems to reduce later memory for the emotion eliciting event (e.g., Sheppes and Meiran, 2007). If the beneficial effect of humor on experienced emotions is based on a cognitive reinterpretation of negative stimuli in less threatening ways (Samson and Gross, 2012), later memory may not be affected by humor because the emotion-eliciting event is still fully attended, an assumption which is supported by the finding that (non-humorous) cognitive reappraisal seems not reduce later memory (Richards and Gross, 2000; Hayes et al., 2010). Finally, it may even be that humor enhances later memory for humorously reappraised negative stimuli. If the beneficial effect of humor on experienced negative emotions is based on an undoing of negative by positive emotions, the evoked positive emotions may bring about an additional boost for memory (e.g., Herbert et al., 2008), an assumption which is supported by findings that humorous material is better remembered than neutral material (e.g., Schmidt, 1994, 2002; Carlson, 2011).

**FIGURE 1:**
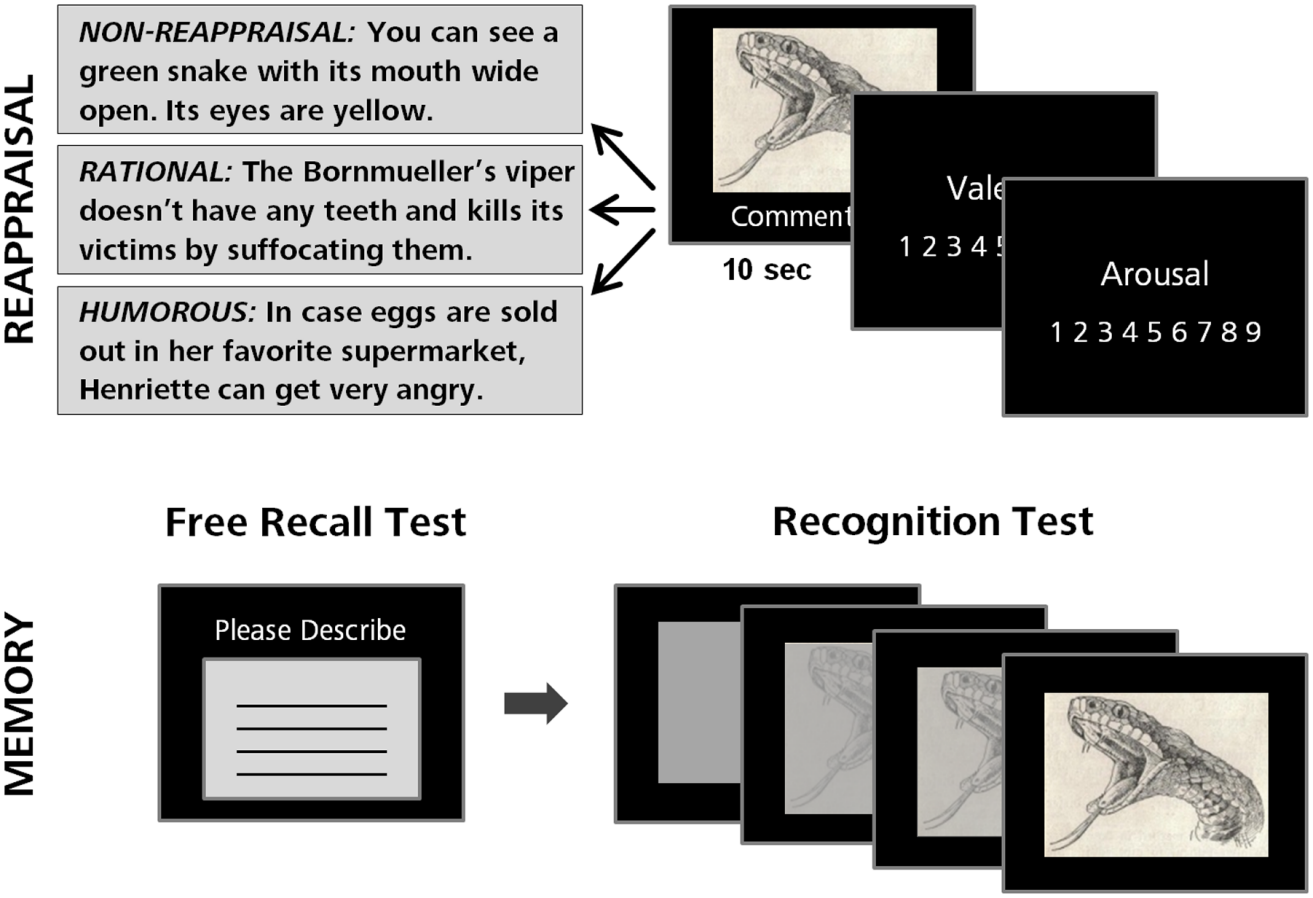
**Procedure of the experiment**. Participants were shown 24 negative and 24 neutral pictures provided with a non-reappraisal control, a rational, or a humorous comment in random order. Each picture was rated on experienced emotional valence and arousal. After picture presentation, a surprise memory test followed. In a first free recall test, participants were asked to verbally describe as many of the previously presented pictures as possible. In a subsequent recognition test, all initially presented pictures were shown again together with 48 new pictures, and participants were instructed to indicate whether a picture was old or new. In order to measure memory strength of recognized pictures, we used a successive disclosure procedure where participants were asked to press a button as soon as they were able to identify a picture as having been shown before. The picture printed here is for example only; to maintain the research value of the images in the International Affective Picture System (IAPS), we have not included actually shown IAPS pictures.

The aim of the present study was to examine the effects of humorous emotion regulation on both current emotional experiences and later memory about emotion-eliciting stimuli. Basically, we followed the procedure introduced by Samson and Gross (2012) and Samson et al. (2014) where participants rate their emotional responses to negative pictures that are shown with the instruction to either simply view the pictures (control condition), rationally reappraise the pictures (rational reappraisal condition), or humorously reappraise the pictures (humorous reappraisal condition). However, in order to overcome a few methodological shortcomings of previous studies, a number of changes were made. First, we included not only emotionally negative pictures but also emotionally neutral pictures in order to be able to examine whether the effects of humorous reappraisal are similar for neutral and negative pictures, or specific to negative pictures. Second, to standardize reappraisal, rather than asking participants to provide individual remarks in the reappraisal conditions, pictures were accompanied by standardized written humorous or rationalizing comments (for examples, see **Figure 1**). Third, in order to control for the potential confounding effect that pictures in the control condition are simply viewed whereas pictures in the reappraisal conditions are additionally verbally processed, pictures in the control condition were shown with a written comment as well that simply described the picture content. After picture presentation, memory for the pictures was assessed both for free recall and recognition memory, in order to obtain a comprehensive view of the effects of humor on later memory.

With respect to the effects of humor on the strength of elicited emotional experiences, we expected that humorous reappraisals should down-regulate evoked negative emotions, replicating findings by Samson and Gross (2012). In particular, based on the recent findings of Samson et al. (2014), we expected that humorous reappraisal should be more effective in down-regulating negative emotions than rational reappraisal because the elicitation of positive emotions involved in humorously reappraisal can help to further “undo” negative emotions beyond the effects of purely rational reappraisal. With respect to the effects of humor on later memory for reappraised stimuli, if the beneficial effects of humor on emotional experiences are mainly based on cognitive distraction, memory performance should be decreased in the humorous reappraisal condition compared to the other conditions. If the beneficial effects of humor on emotional experiences are based on cognitive reappraisal, memory performance should be similar between the humorous and rational reappraisal conditions, and according to the findings by Richards and Gross (2000), memory performance in the reappraisal conditions should be comparable to the control condition. If the beneficial effects of humor on emotional experiences are based on an undoing of negative by positive emotions, memory performance in the humorous reappraisal condition may even be increased compared to the other conditions.

## Materials and Methods

### Participants

To detect small-sized effects (*d* = 0.4, α = 0.05) with sufficient power (0.80), a sample size of 52 is required. Therefore, we planned to collect data from at least 52 participants until the end of the semester. This resulted in a sample of 63 undergraduate students (45 females, mean age = 24.92 years, SD = 4.61), who participated for course credit. Each person was tested individually. The study was conducted in accordance with the Helsinki Declaration and the University Research Ethics Standards.

### Materials

Twenty-four neutral and twenty-four negative pictures were selected. Most of the pictures were drawn from the International Affective Picture System (IAPS; Lang et al., 1999), additionally, three of the neutral pictures were taken from the Geneva affective picture database (GAPED; Dan-Glauser and Scherer, 2011). Pictures were chosen by the criteria of reasonableness and differentiability, and every negative picture was yoked with a visually similar neutral picture. Negative pictures were selected to be more negatively valenced and more arousing than neutral pictures (Valence: *M*_Negative_ = 2.84, SD = 0.65; *M*_Neutral_ = 5.33, SD = 0.52; Arousal: *M*_Negative_ = *M* = 5.59, SD = 0.80; *M*_Neutral_ = 3.79, SD = 1.01).

For each picture, a humorous, a rationalizing, and a neutral comment were generated (all comments are provided as Supplementary Material; for examples, see **Figure 1**). The humorous comments reflected a positive form of humor in the sense of Samson and Gross (2012) and were generated according to their instructions (i.e., reappraising in a benevolent and amusing way without becoming hostile or aggressive, focusing on absurdities of situations). The rationalizing comments reflected a rational form of cognitive reappraisal in the sense of Richards and Gross (2000), and were generated according to their instructions (i.e., adopting a neutral attitude when watching a picture by thinking about it objectively and analytically); the non-reappraisal comments verbally described what could be seen on the picture. The three types of comments were matched on the number of words (*M*_Humorous_ = 14.52, *M*_Rational_ = 14.56, *M*_Neutral_ = 13.96).

### Design and Procedure

A 2 × 3 within-subject design was used with the factors of emotional content of a picture (neutral vs. negative) and reappraisal condition (humorous vs. rational vs. neutral). The participants were shown the 24 neutral and 24 negatives pictures on a computer screen in random order using E-Prime 2.0 (PST, Pittsburgh, PA, USA) with the instruction to rate their emotional responses to each picture on valence (1 = extremely negative to 9 = extremely positive) and arousal (1 = not at all aroused to 9 = extremely aroused). No mention was made that memory for the pictures will be tested later. One third of the neutral, respectively, negative, pictures were shown with a humorous comment, one third with a rationalizing comment, and one third with a neutral comment. The assignment of type of comments to the pictures was counterbalanced across participants.

Each picture was shown for 10 s at the center of the screen with the comment displayed below the picture (see **Figure 1**). Participants were instructed to look at the pictures as long as they were presented and to read the respective comments carefully. After presentation of each picture, the valence and arousal scales were shown and participants made their ratings without any time restriction. After the presentation of all 48 pictures, a 1-min distractor phase followed in which participants had to solve simple arithmetic problems. A surprise free recall test for the presented pictures followed, in which participants were instructed to verbally describe on a provided sheet as many of the previously presented pictures as could be recalled any time restriction. After another 1-min distractor phase, a surprise recognition memory test followed. Participants were shown all initially presented pictures again together with 48 new pictures (24 negative and 24 neutral pictures, taken from the IAPS and GAPED data bases) in random order. In order to measure not only general recognition memory in an all-or-none fashion but also assess the memory strength of recognized pictures, we used a successive disclosure procedure. Each picture was presented in 100 gradation slides in ascending order, starting with a completely gray slide until the picture was entirely visible. Each gradation slide was shown for 66 ms so that the picture sequence appeared as a continuum. Participants were asked to press a button as soon as they were able to identify a picture as having been shown before. If the disclosed picture was judged to be new, they were asked to wait until the picture was fully visible without pressing any button. General recognition memory was measured as the proportion of correctly recognized pictures independently of when the button was pressed during the disclosure sequence, and memory strength was measured as the time necessary for correctly recognizing a previously presented picture.

## Results

### Elicited Emotions

**FIGURE 2:**
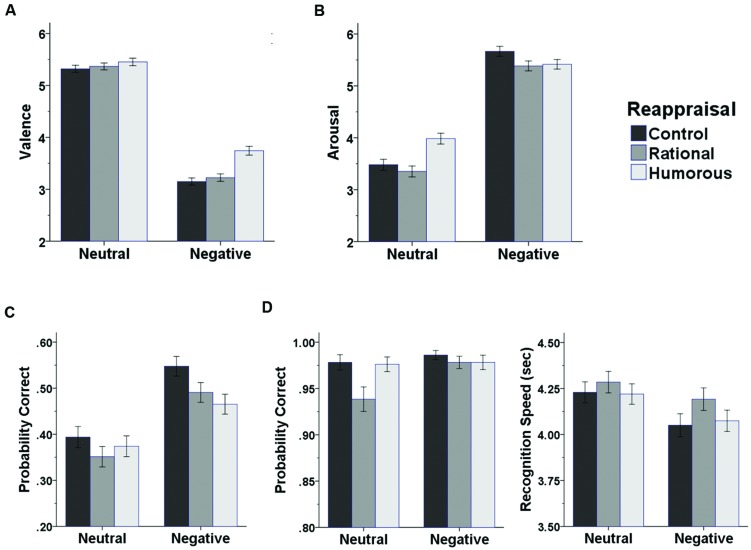
**Results of the experiment**. (A) Emotional valence ratings (1 = extremely negative to 9 = extremely positive), **(B)** emotional arousal ratings (1 = not at all aroused to 9 = extremely aroused), **(C)** free recall performance, and **(D)** recognition performance as a function of emotional content of pictures (neutral, negative) and reappraisal condition (control, rational, humorous). The left panel in **(D)** shows recognition accuracy (probability of correct recall), the right panels shows the time needed to correctly identify a previously presented. Error bars represent SE.

**Figure 2** shows valence (**Figure 2A**) and arousal (**Figure 2B**) ratings of participants as a function of emotional content of pictures and reappraisal condition. To analyze the effect of type of reappraisal on emotional experiences, we conducted analyses of variances (ANOVA) for valence and arousal ratings with factors of emotional content of pictures (neutral vs. negative) and reappraisal condition (humorous vs. rational vs. control). For valence, there was a significant main effect of emotional content of pictures, *F*(1,62) = 152.62, *p* < 0.001, $\eta_{p}^{2}$ = 0.71, indicating that negative pictures were much more negatively experienced than neutral pictures. There was also a significant main effect of reappraisal condition, *F*(2,124) = 14.94, *p* < 0.001, $\eta_{p}^{2}$ = 0.19, indicating that experienced negativity varied as a function of type of reappraisal. The interaction between both factors was also significant, *F*(2,124) = 10.60, *p* < 0.001, $\eta_{p}^{2}$ = 0.15, indicating that the differential effects of reappraisal type differed between neutral and negative pictures. For negative pictures, humorous reappraisal increased valence ratings compared to both the control condition, *t*(62) = 6.40, *p* < 0.001, *d* = 0.81, 95% CI: 0.43 to 0.81, and the rational reappraisal condition, *t*(62) = 5.84, *p* < 0.001, *d* = 0.74, 95% CI: 0.35 to 0.72; valence ratings did not significantly differ between the rational reappraisal and control conditions, *t*(62) = 1.07, *p* = 0.290, *d* = 0.13, 95% CI: –0.07 to 0.25 For neutral pictures, valence ratings did not significantly differ between conditions, *F*(2,124) = 1.02, *p* = 0.364, $\eta_{p}^{2}$ = 0.02.

For arousal, there also were significant main effects of emotional content of pictures, *F*(1,62) = 97.35, *p* < 0.001, $\eta_{p}^{2}$ = 0.61, and reappraisal condition, *F*(2,124) = 5.96, *p* = 0.003, $\eta_{p}^{2}$ = 0.09, and a significant interaction between both factors, *F*(2,124) = 20.86, *p* < 0.001, $\eta_{p}^{2}$ = 0.25. For negative pictures, both humorous and rational reappraisal decreased arousal ratings, compared to the control condition, *t*(62) = –2.71, *p* = 0.009, *d* = 0.34, 95% CI: –0.46 to –0.07, and *t*(62) = –2.86, *p* = 0.006, *d* = 0.36, 95% CI: –0.45 to –0.08, respectively; arousal ratings between the humorous and rational reappraisal conditions did not differ, *t*(62) = 0.00, *p* = 0.999, *d* = 0.00, 95% CI: –0.20 to 0.20. For neutral pictures, humorous reappraisal increased arousal ratings compared to both the control condition, *t*(62) = 3.43, *p* = 0.001, *d* = 0.43, 95% CI: 0.20 to 0.77, and the rational reappraisal condition, *t*(62) = 4.67, *p* < 0.001, *d* = 0.59, 95% CI: 0.36 to 0.94, whereas arousal ratings did not significantly differ between the rational reappraisal and control conditions, *t*(62) = –1.55, *p* = 0.126, *d* = 0.20, 95% CI: –0.39 to 0.05.

### Memory Performance

#### Free Recall

**Figure 2C** shows free recall memory performance as a function of emotional content of pictures and reappraisal condition. An ANOVA with factors of emotional picture content (neutral vs. negative) and reappraisal condition (neutral vs. humorous vs. rational) revealed a significant main effect of picture content, *F*(1,62) = 70.84, *p* < 0.001, $\eta_{p}^{2}$ = 0.53, indicating that negative pictures were much better remembered than neutral pictures. There was also a significant main effect of reappraisal condition, *F*(2,124) = 4.22, *p* = 0.017, $\eta_{p}^{2}$ = 0.06, indicating that memory performance varied as a function of type of reappraisal. The interaction between both factors was not significant, *F*(2,124) = 0.66, *p* = 0.518, $\eta_{p}^{2}$ = 0.01. Overall, compared to the control condition, both humorous (*M*_Decrease_ = –5.49%) and rational reappraisal (*M*_Decrease_ = –5.37%) decreased memory performance, *t*(62) = –2.35, *p* = 0.022, *d* = 0.30, 95% CI: –0.10 to -0.01, and *t*(62) = –2.98, *p* = 0.004, *d* = 0.38, 95% CI: –0.09 to –0.02, respectively. Amount of decrease did not differ between the humorous and rational reappraisal conditions, *t*(62) = –0.05, *p* = 0.961, *d* = 0.01. Analyzing data separately for negative and neutral pictures revealed that for negative pictures, memory performance was decreased both in the humorous and rational reappraisal conditions, compared to the control condition, *t*(62) = –2.51, *p* = 0.015, *d* = 0.31, 95% CI: –0.14 to –0.02, and *t*(62) = –2.12, *p* = 0.038, *d* = 0.27, 95% CI: –0.11 to 0.00, respectively. For neutral pictures, memory performance did not significantly differ between conditions, *F*(2,124) = 1.20, *p* = 0.304, $\eta_{p}^{2}$ = 0.02.

#### Recognition

The false alarm rate was very low and did not vary as a function of emotional contents of lures (*M*_Negative_ = 2.05%, SD = 2.89; *M*_Neutral_ = 1.59%, SD = 2.84), *t*(62) = 1.12, *p* < 0.266, *d* = 0.14, 95% CI: 0.00 to 0.13. **Figure 2D** (left) shows the proportion of correctly recognized pictures as a function of emotional content of pictures and reappraisal condition. An ANOVA with factors of emotional picture content (neutral vs. negative) and reappraisal condition (neutral vs. humorous vs. rational) revealed a significant main effect of picture content, *F*(1,62) = 5.44, *p* = 0.023, $\eta_{p}^{2}$ = 0.08, indicating that negative pictures were better recognized than neutral pictures. There was also a significant main effect of reappraisal condition, *F*(2,124) = 5.89, *p* = 0.004, $\eta_{p}^{2}$ = 0.09, indicating that recognition memory performance varied as a function of type of reappraisal. The interaction between both factors was also significant, *F*(2,124) = 3.62, *p* = 0.030, $\eta_{p}^{2}$ = 0.06, indicating that the differential effects of reappraisal type differed between neutral and negative pictures. For negative pictures, recognition memory performance did not significantly differ between conditions, *F*(2,124) = 0.53, *p* = 0.590, $\eta_{p}^{2}$ = 0.01. For neutral pictures, rational reappraisal decreased recognition memory performance compared to both the humorous condition, *t*(62) = –2.93, *p* = 0.005, *d* = 0.37, 95% CI: –0.06 to –0.01, and the control reappraisal condition, *t*(62) = –2.87, *p* = 0.006, *d* = 0.36, 95% CI: –0.07 to –0.01; recognition memory performance did not significantly differ between the humorous reappraisal and control conditions, *t*(62) = –0.241, *p* = 0.811, *d* = 0.03, 95% CI: –0.02 to 0.01.

**Figure 2D** (right) shows the time necessary for correctly recognizing a previously presented picture, reflecting underlying memory strength, as a function of emotional content of pictures and reappraisal condition. An ANOVA with factors of emotional picture content (neutral vs. negative) and reappraisal condition (neutral vs. humorous vs. rational) revealed a significant main effect of picture content, *F*(1,62) = 28.37, *p* < 0.001, $\eta_{p}^{2}$ = 0.31, indicating that negative pictures were more quickly recognized than neutral pictures. There was also a significant main effect of reappraisal condition, *F*(2,124) = 3.07, *p* = 0.050, $\eta_{p}^{2}$ = 0.05, indicating that recognition speed varied as a function of type of reappraisal. The interaction between both factors was not significant, *F*(2,124) = 0.53, *p* = 0.591, $\eta_{p}^{2}$ = 0.01. In the rational reappraisal condition, recognition speed was decreased compared to both the humorous condition, *t*(62) = –1.96, *p* = 0.054, *d* = 0.25, 95% CI: –2.79 to 0.03, and the control condition, *t*(62) = –2.43, *p* = 0.018, *d* = 0.31, 95% CI: –2.72 to –0.26. Recognition speed did not differ between the humorous and control conditions, *t*(62) = 0.16, *p* < 0.873, *d* = 0.02, 95% CI: –1.27 to 1.49.

## Discussion

In the present study, we investigated whether humor may be a functional strategy to regulate negative emotions by examining the effects of humorous reappraisal compared to rational reappraisal and non-reappraisal on evoked emotional experiences and later memory for the emotion-eliciting stimuli. The results showed that humor seems to be indeed an optimal strategy to adaptively cope with stimuli that elicit negative emotions. Regarding evoked emotional experiences, humorous reappraisal reduced experienced negative valence and arousal, replicating previous findings (Samson and Gross, 2012). Thus, humor can indeed help us to feel better when being confronted with negative events. In particular, replicating the recent findings by Samson et al. (2014), our results showed that humorous reappraisal is more successful in down-regulating negative emotions than rational reappraisal because rational reappraisal reduced only arousal levels but not experienced negative valence.

Regarding later memory for emotion-eliciting stimuli, the results showed that humorous reappraisal reduced free recall for negative stimuli compared to non-reappraisal, indicating that humor reduces the presence of previously experienced negative events in mind when actively reconstructing our past. However, the results for the recognition test showed that at the same time recognition memory for negative stimuli was completely intact in the humorous reappraisal condition, indicating that emotion-eliciting events were still fully stored in memory. From a functional perspective, such a pattern seems to be adaptive because on the one hand, undergone negative experiences less strongly infiltrate our minds when remembering our past in contexts that do not match the previous emotion-eliciting situation. On the other hand, however, when the contextual information matches the features of the previous emotion-eliciting situation, then past experiences can nevertheless be fully retrieved in order to prepare for appropriate responding. In particular, similar to the effects on elicited emotional experiences, humorous reappraisal seems to be even more functional than rational reappraisal because rational reappraisal did not only reduce free recall but also reduce the strength of recognition memory.

There is still a debate on whether the effectiveness of humor as an emotion regulation strategy is attributable to the mechanisms of cognitive distraction from negative stimuli (e.g., Strick et al., 2009), or to cognitive reappraisal of negative stimuli in less threatening ways (e.g., Samson and Gross, 2012). Previous research has shown that distraction and reappraisal differ with respect to the consequences for later memories about the emotion-eliciting event, with distraction, but not reappraisal, impairing later recognition memory (e.g., Richards and Gross, 2000; Sheppes and Meiran, 2007). Thus, the finding of the present study that humorous reappraisal did not impair recognition memory strongly supports the view that the mechanism underlying humor as an emotion regulation strategy is reappraisal.

Indeed, such a view is further supported by the finding that humorous reappraisal differentially affected recognition memory and free recall. Whereas humor did not influence recognition memory, free recall was impaired. Such a pattern speaks against the assumption that distraction may underlie the effects of humorous reappraisal because previous research has shown that cognitive distraction during encoding impairs both free recall and recognition memory (e.g., Craik et al., 1996). Instead, such differential effects on recognition and free recall support the assumption that the effect of humor is based on cognitive reappraisal. One factor which is known to differentially influence free recall and recognition is whether processing during encoding is focused on the relationship between a stimulus and other stimuli (i.e., relational processing), or on the individual characteristics of a stimulus (i.e., item-specific processing). Whereas item-specific processing reduces free recall because the memory representation of a stimulus is less strongly activated by other stored stimuli so that active reproduction is impaired, item-specific processing does not impair recognition memory because an active reproduction of the to-be-remembered stimulus is not necessary for recognition (e.g., Einstein and Hunt, 1980; Engelkamp et al., 1998). Thus, as the attempt to reappraise a stimulus in a humorous way requires focusing on the to-be-reappraised stimulus, the underlying mechanism of the effects of humor on memory seems to be the induction of item-specific processing.

In fact, a similar mechanism may explain the effect of rational reappraisal on memory. Replicating previous findings, recognition accuracy for negative pictures was not impaired by rational reappraisal (Richards and Gross, 2000; Hayes et al., 2010). However, going beyond previous findings, the present results demonstrate that free recall is impaired. Thus, similar to humorous reappraisal, rational reappraisal seems also to induce an item-specific processing of the to-be-reappraised stimuli, leading to the observed differential effects on later free recall and recognition memory. However, with respect to the effects on elicited emotions, the results indicate that cognitive reappraisal alone is less effective in down-regulating negative emotions than when the cognitive reappraisal additionally evokes positive emotions due to a humorous reinterpretation of stimuli. Thus, an evoking of positive emotions, as induced by humorous reappraisal, seems to be necessary to really undo experienced negative emotions (e.g., Fredrickson et al., 2000). However, the undoing of negative by humor-induced positive emotions seems not to be strong enough to bring about an additional boost in memory.

One interesting finding of the present study is that rational reappraisal was rather ineffective in down-regulating negative emotions because only elicited emotional arousal but not negative valence was reduced. On first glance, such a finding seems to deviate from previous studies showing decreased valence ratings when rationally reappraising compared to when simply watching emotion-eliciting stimuli in a non-reappraisal control condition (e.g., Richards and Gross, 2000; Hayes et al., 2010). However, a closer look reveals that there is one important difference between the present and the previous studies. In previous studies, reappraisal and control conditions differed not only in terms of reappraisal but also in terms of cognitive processing in general because participants in the control condition were instructed to simply watch the pictures, whereas in the reappraisal condition additional cognitive processing was required. In the present study, the control and reappraisal conditions were matched on required cognitive processing in order to control for the potential confounding effect of cognitive processing in general. The finding that the benefits from rational reappraisal were rather small under such conditions suggest that the down-regulating of negative emotions found in previous studies may more likely reflect the effect of cognitive processing in general, rather than specific effects of rational reappraisal. Indeed, such an assumption is supported by recent findings showing that additional cognitive processing during the perception of emotional events can reduce negative emotions (e.g., Van Dillen and Koole, 2007; Strick et al., 2009). However, given that the present study did not include a condition where emotional stimuli were simply watched, further research is needed to clarify the specific effects of rational reappraisal beyond the effects of cognitive processing in general.

In the present study, the form of reappraisal employed in the rational reappraisal condition reflected a rational form of cognitive reappraisal where emotion regulation is based on the attempt to adopt a neutral attitude when watching a picture by thinking about it objectively and analytically (e.g., Richards and Gross, 2000). However, there are other forms of cognitively reappraising emotionally negative situations, such as trying to think about a situation in a more positive light, or thinking about the positive bearing an event could have on the persons involved in the situation (e.g., Troy et al., 2010). One important difference between these different forms of cognitive reappraisals is that the latter one may additionally elicit positive emotions due to the thinking about potential positive aspects of the given negative situation. Thus, it may be that such “positive” forms of cognitive reappraisal may be similar effective than humorous reappraisal where the elicitation of positive emotions seems to play an important role as well, an open question that warrants future research.

## Conclusion

Humor seems to be indeed an especially functional emotion regulation strategy that can outperform other emotion regulation strategies such as rational reappraisal. Thus, Freud (1905/1960) may have been right in assuming that humor can be seen as the most valuable high-level defense of unpleasure.

## Conflict of Interest Statement

The authors declare that the research was conducted in the absence of any commercial or financial relationships that could be construed as a potential conflict of interest.

## References

[B1] Carlson K. A. (2011). The impact of humor on memory: is the humor effect about humor? *Humor* 24 21–41. 10.1515/humr.2011.002

[B2] Craik F. I. M. Govoni R. Naveh-Benjamin M. Anderson N. D. (1996). The effects of divided attention on encoding and retrieval processes in human memory. *J. Exp. Psychol. Gen.* 125 159–180. 10.1037/0096-3445.125.2.159 8683192

[B3] Dan-Glauser E. S. Scherer K. R. (2011). The Geneva affective picture database (GAPED): a new 730-picture database focusing on valence and normative significance. *Behav. Res. Methods* 43 468–477. 10.3758/s13428-011-0064-1 21431997

[B4] Einstein G. O. Hunt R. R. (1980). Levels of processing and organization: additive effects of individual-item and relational processing. *J. Exp. Psychol. Learn.* 10 133–143. 10.1037/0278-7393.6.5.588

[B5] Engelkamp J. Biegelmann U. McDaniel M. A. (1998). Relational and item-specific information: trade-off and redundancy. *Memory* 6 307–333. 10.1080/741942360 9709445

[B6] Fredrickson B. L. Mancuso R. A. Branigan C. Tugade M. M. (2000). The undoing effect of positive emotions. *Motiv. Emot.* 24 237–258. 10.1023/A:1010796329158 21731120 PMC3128334

[B7] Freud S. (1905/1960). *Jokes And Their Relation To The Unconscious* (Strachey J. Trans.). New York, NY: W. W. Norton.

[B8] Freud S. (1928). Humour. *Int. J. Psychoanal.* 9 1–6.

[B9] Frijda N. H. (1988). The laws of emotion. *Am. Psychol.* 43 349–358. 10.1037/0003-066X.43.5.349 3389582

[B10] Hamann C. (2001). Cognitive and neural mechanisms of emotional memory. *Trends. Cogn. Sci.* 5 394–400. 10.1016/S1364-6613(00)01707-1 11520704

[B11] Hayes J. P. Morey R. A. Petty C. M. Seth S. Smoski M. J. McCarthy G. M. (2010). Staying cool when things get hot: emotion regulation modulates neural mechnanisms of memory encoding. *Front. Neurosci.* 4:230 10.3389/fnhum.2010.00230 PMC3015134 21212840

[B12] Herbert C. Junghöfer M. Kissler J. (2008). Event related potentials to emotional adjectives during reading. *Psychophysiology* 45 487–498. 10.1111/j.1469-8986.2007.00638.x 18221445

[B13] Lang P. J. Bradley M. M. Cuthbert B. N. (1999). *International Affective Picture System (IAPS): Technical Manual And Affective Ratings*. Gainesville: University of Florida, Center for Research in Psychophysiology.

[B14] Mulligan N. W. (2008). “Attention and memory,” in *Learning and Memory: A Comprehensive Reference* ed. Roediger H. L. (Oxford: Elsevier) 7–22. 10.1016/B978-012370509-9.00134-0

[B15] Richards J. M. Gross J. J. (2000). Emotion regulation and memory: the cognitive costs of keeping one’s cool. *J. Pers. Soc. Psychol.* 79 410–424. 10.1037/0022-3514.79.3.410 10981843

[B16] Samson A. C. Glassco A. Lee I. A. Gross J. J. (2014). Humorous coping and serious reappraisal: short-term and longer-term effects. *Eur. J. Psychol.* 10 571–581. 10.5964/ejop.v10i3.730

[B17] Samson A. C. Gross J. J. (2012). Humour as emotion regulation: the differential consequences of negative versus positive humour. *Cogn. Emot.* 26 375–384. 10.1080/02699931.2011.585069 21756218

[B18] Schmidt S. R. (1994). Effects of humor on sentence memory. *J. Exp. Psychol. Learn.* 20 953–967. 10.1037/0278-7393.20.4.953 8064254

[B19] Schmidt S. R. (2002). The humour effect: differential processing and privileged retrieval. *Memory* 10 127–138. 10.1080/09658210143000263 11798442

[B20] Sheppes G. Meiran N. (2007). Better late than never? On the dynamics of online regulation of sadness using distraction and cognitive reappraisal. *Pers. Soc. Psychol. Bull.* 33 1518–1532. 10.1177/0146167207305537 17933748

[B21] Strick M. Holland R. W. van Baaren R. B. van Knippenberg A. (2009). Finding comfort in a joke: consolatory effects of humor through cognitive distraction. *Emotion* 9 574–578. 10.1037/a0015951 19653782

[B22] Troy A. S. Wilhelm F. H. Shallcross A. J. Mauss I. B. (2010). Seeing the silver lining: cognitive reappraisal ability moderates the relationship between stress and depression. *Emotion* 10 783–795. 10.1037/a0020262 21058843 PMC3278301

[B23] Van Dillen L. F. Koole S. L. (2007). Clearing the mind: a working memory model of distraction from negative mood. *Emotion* 7 715–723. 10.1037/1528-3542.7.4.715 18039038

