# Supplementary material for: That’s not funny! – But it should be: effects of humorous emotion regulation on emotional experience and memory
Source: Front Psychol. 2015 Aug 28;6:1296. doi: 10.3389/fpsyg.2015.01296 (PMC4551820; doi:10.3389/fpsyg.2015.01296)
Supplement: Supplementary file 1 [file Table_1.DOCX]

Supplementary Material

**That’s Not Funny! – But It Should Be: Effects of Humorous Emotion Regulation on Emotional Experience and Memory**

**Lisa Kugler*, Christof Kuhbandner**

*** Correspondence:** Lisa Kugler: lisa.kugler@ur.de

# Supplementary Figures and Tables

| **Supplementary Table 1.** Comments (English Translation of the original German comments) for each of the presented pictures (IAPS and GAPED) in the non-reapraisal, the humorous reappraisal, and the rational reappraisal conditions. Each negative picture was yoked with a thematically similar but neutral picture; pictures that belong together are listed consecutively. | | | |
| --- | --- | --- | --- |
| **IAPS/GAPED picture** | **Non-reappraisal comment** | **Humorous comment** | **Rationalizing comment** |
| **1201 (spider)** | A big spider is sitting on a men’s shoulder who is wearing a white t-shirt. | That is the outcome when you buy a parrot in the internet instead of going to a pet shop. | In Europe around 1.300 spider species exist. Two of them are toxic for human beings. |
| **1450 (gannet)** | A white bird is sitting on a stone and is illuminated by a front side light. | Erna will probably never accept that she doesn’t get suntanned. | The gannets (sulidae) are part of the family of seabirds in the category of totipalmate swimmers (pelicaniformes). |
| **1247 (cockroaches)** | Two small and two big cockroaches are lying side by side on a blue base. | Lucky Luke is needed urgently: Since yesterday the Daltons have been making trouble again. | The female African Desert Cockroach is typically by one third larger than a male. |
| **1390 (bees)** | You can see six bees whose wings reflect the light coming from above. | Maja and Willi have been standing in the line for the ACDC concert for a whole hour now. | In Europe you can find nearly 700 bee species, of which 500 of them are located in Germany. |
| **1300 (dog with bared teeth)** | You can see the head of a brown dog with an offensively open mouth. | The dentist is satisfied and thinks that the braces have been doing a good job for Emil. | The permanent dentition of a dog consists of 42 teeth. There are 3 incisors in both parts of the jaw. |
| **1670 (cow)** | A black cow with a few white spots is standing on the grass looking into the camera. | Even if it sounds surprisingly for city kids: Cows are not purple and white. | The common cattle (lat. bos primigenius baurus) is the domesticated relative of the aurochs. |
| **1050 (green snake)** | You can see a green snake with its mouth wide open. Its eyes are yellow. | In case eggs are sold out in her favorite supermarket, Henriette can get very angry. | The Bornmueller’s viper doesn’t have any teeth and kills its victims by suffocating them. |
| **1945 (turtle with worm)** | A turtle having an earthworm in its mouth is sitting on the ground looking into the camera. | The worm was very happy to have managed to escape from the early bird. | Turtles are basically omnivore. Depending on the species they tend to be carnivore or herbivorous, though. |
| **2120 (angry looking man)** | A man in a black pullover is facing the camera with widely opened eyes and mouth. | He literally meant that the itchy woolen pullover would drive him crazy sooner or later. | Abnormally increased sweating which can occur locally or globally is called hyperhidrosis. |
| **2220 (a man’s eyes)** | A man with light brown hair is touching the area around his eyes with two fingers. | Hans has mistaken the superglue for his wife’s skin tightening lotion, which he wanted to try in secret. | Only few people can determine precisely whether the area around the eyes belongs to a male or a female face. |
| **2205 (man sitting on a woman’s bed)** | An old man is sitting next to his wife’s bed, holding her hand. | Having a severe hangover has always been horrible for Rudi and Berta. | Compared to former times the differences in life expectancy between men and women have decreased. |
| **GAPED N066 (man and woman working)** | A man is holding an object in his hands and a woman is sitting in front of a computer. | In times of digitalization parents can tell the Easter bunny precisely what kind of Easter basket they want to have. | A recently developed software for archeological excavation offers new ways of documentation. |
| **2691 (throwing man)** | A man is about to throw a stone. In the background there are several other people. | The highlight of the meeting was the legendary target tossing. As always the first price is a kiss from the festival princess. | In Germany, demonstrations are only forbidden if they endanger security and public order. |
| **2880 (shadow)** | One can see the shadow of a man on the street who is spreading his arms. | Every time after watching “Titanic” he considers himself to be the king of the world. | A shadow is an area where light from a light source is obstructed by an opaque object. |
| **2692 (bomb)** | The thumb of a man’s hand is resting on the red button of a bomb’s ignition. | Peter has built an alarm clock out of sausages that will smell like salami at a set time. | Depending on its size and explosive power, dynamite causes damages of varied expenses. |
| **GAPED N065 (spiral)** | Two hands are holding a plastic spiral from both ends. | Here you can see a typical earth snake. Unfortunately, it dried out during the last heatwave. | Ebonite is an especially robust rubber made of natural resources (natural rubber and sulfur). |
| **2710 (drug addict)** | A man is sitting on the floor with closed eyes, holding a syringe in his hand. | Hans-Jürgen came back extremely relaxed from his last wellness trip to Tijuana. | Heroin is a partly synthetic, strongly analgetic opioid with a high addictive potential. |
| **2749 (men smoking cigar)** | A man with a grey shirt is sitting at a table in front of a cupboard, smoking a cigar. | Having such distasteful furniture probably everyone would start consuming alcohol and drugs. | The best known cigar is called “Corona”. It is 140mm long and has a diameter of about 16mm. |
| **2750 (old man drinking)** | A dirty man wearing a hat and dark clothes is drinking from a green bottle. | During his wellness days, drinking enough liquids and keeping a healthy diet are very important for him. | In 2012 there were about 284.000 homeless people in Germany. |
| **2221 (judge)** | A judge is sitting at his table in the courtroom, facing the camera. | It would have been nice for Gisbert, if someone had explained to him that studying law doesn’t qualify him to be an auctioneer. | After completing law school, one has to do a traineeship for two years before becoming a judge. |
| **3301 (wounded child)** | One can see the bandaged face of an injured child which is touched by two hands. | The napkin wasn’t enough. Klausi was full of jam from head to toe. | About a quarter of the patients in the trauma surgery in Innsbruck are children and teenagers. |
| **2840 (child with chessboard)** | A boy is sitting in front of a chess board, leaning his head on his right hand. | Instead of playing chess, Timmy wanted to make a mess. His hearing impaired grandmother must have got that wrong. | Playing chess supports power of deduction and logical reasoning. This can have a positive impact on a child’s development. |
| **3530 (man threatening another man)** | A man with white hair and a black cap is pointing towards a bleeding person with his gun. | Recently, a ticket inspector has started to work in public transportation which is a bit drastic when it comes to dealing with customers. | The US Colt 1911 Government is the pistol which was used by the ordinances of the US armed forces from 1911 until 1985. |
| **2580 (men playing chess)** | Two elderly men are sitting outside at a table playing chess. | Mr. Etsabakis and Mr. Rosenbaum met every afternoon in the Central Park for having a short nap. | A professionally played chess match (following the ordinary rules) can last for six hours or longer. |
| **5971 (hurricane)** | In the foreground one can see a city with buildings. In the background there is a whirlwind. | Perfect wind is exactly what the brochure has promised them for their sailing trip. | In Germany, tornados are called “Großtromben”, “Windhosen” or “Wasserhosen”. |
| **5900 (dry landscape)** | On an unpaved road beneath the blue sky and in front of the mountains are two bushes. | When they booked a calm trip in the travel agency, “Wool” and “Ball” expected something different. | Australia has a lot of paved roads. In the outback, lots of roads are unpaved, though. |
| **6010 (man behind bars)** | A prisoner in a white t-shirt is holding documents in his hand and looking through the bars. | In case flirting with the house lady is successful, William sometimes remits the bill for the garden gate. | Prisoners who are prematurely discharged from jail are less likely to commit another crime than prisoners who have served the full sentence. |
| **2514 (red house)** | An elderly lady with grey hair and glasses is looking out of the window of a red house. | As she has taken the wrong night train, Julia is waiting for Romeo in Prague for decades now. | This picture shows the “Hotel Liliova” in Prague which is a historical city mansion from the 18^th^ century. |
| **6020 (electric chair)** | A wooden electric chair is standing on a black floor in the edge of a room. | Thanks to modern technology, electric chairs help to prevent the dying from hurting themselves by falling. | The execution protocol in Florida contains precise regulations concerning the strength and duration of the electric shocks. |
| **7235 (chair)** | A brown, wooden chair is standing on a tiled floor, the back pointing towards the blue wall. | The chair “Woody” is upset. He would have preferred being a beautiful table. | The perfect height of a chair varies between 42 and 48 cm depending on the body height. |
| **6370 (masked man)** | One can see the head of a masked person on a blurred picture. | The SUN reveals: The Phantom of the Opera was arrested by mistake as it wanted to draw money from the bank. | The ban on wearing masks forbids protesters to hide their identities during demonstrations. |
| **2190 (man with hat)** | A middle-aged man with a grey cap and a grey collar is facing the camera. | Styling tips: A crooked cap corrects a crooked face. | High-key pictures underline certain parts of the face of the portrayed person. |
| **9320 (blocked toilet)** | Next to a blocked toilet one can see a red garbage bin. | Well, Karsten didn’t know what soy-ragout was when Sarah asked him to put it in the pot. | Leftovers in the wastewater may attract rats. Nonreturn flaps prevent these animals from entering the waste pipes. |
| **GAPED N106 (toilet tank)** | The picture shows a white toilet tank with metal elements on a tiled wall. | The camouflage of webcams has definitely improved within the last years. | Typically, restrooms are equipped with flushing valves. Frequently in airplane toilets vacuum-flushing-systems are used. |
| **8231 (wounded boxer)** | A bleeding boxer in yellow shorts is lying on the ground. In the background one can see cheering spectators. | His wife Xena did warn him several times not to leave the toothpaste open. | A boxer would be knocked out, if he is unable to continue the fight after a ten second recovery break. |
| **2575 (man with airscrew)** | A man with a moustache and a blue shirt is standing behind a coppery airscrew holding on to it. | Fernando was convinced that this little detail would bring out the best of his Smart. | Airscrews absorb the mechanical work and release it as flow energy. |
| **6838 (screaming girl)** | A girl with its mouth open is standing in front of a white car, two people are lying on the ground. | Susi’s parents stumbled with some McDonald’s bags in their hands. Annoying: She was sooo hungry! | The testimonies of eye-witnesses are extremely unreliable. Young children and elderly people remember details most unreliably. |
| **2870 (boy sitting on an engine bonnet)** | A juvenile is eating while lying on an engine bonnet. Next to him is a radio set. | Protection from hearing defect and sunburn? Easy going: An apple a day keeps the doctor away. | Radio sets had their golden age in the seventies and eighties and were often made in a grandiose style. |
| **9050 (plane crash)** | A crowd of people is moving away from an airplane which tailpiece is emitting black smoke. | Disappointed faces among the holiday-makers: but if they had been travelling with “Deutsche Bahn”, they would have been delayed too. | According to statistics the probability of surviving a crash-landing while takeoff or landing is about 95.7 %. |
| **7620 (plane)** | On this photo passengers use stairs to get into a blue-white-striped airplane during twilight. | The airplane took off with a tremendous delay. The reason: Manfred thought the gangway was an escalator. | Yearly around 5 billion passengers use a plane to get to their travel destination. |
| **9180 (wounded seals)** | Two fully grown seals are lying on a light subsoil. One of them is hurt. | For days Erna has a stitch. Maybe she should scale down her jogging. | At the north coast of Canada seals get killed for gaining fur, oil, and only recently meat as well. |
| **1313 (frog)** | A frog is sitting above the eye of a dog, which can be seen on the picture. | Erwin, the tree frog has always liked trampling on other people. | The European tree frog is an amphibian frog belonging to the category “frogs in a wider sense”. |
| **9421 (crying soldier)** | An injured, crying soldier with a sling is sitting next to another one with a bounded face. | For hours Heinz has been looking desperately for his tissue. His mate hasn’t seen any either. | Out of 19.450 Australians fighting in Vietnam war between 1965 and 1972, 200 got killed and 1.297 injured. |
| **2487 (musician)** | A man with long hair, hat and sunglasses is playing the guitar in a street. | One day his income as a street musician will be sufficient to buy himself a new hat. | Street music has been existing since the early ancient times as we know from vagabond singers from the prehomeric era. |
| **9560 (bird in oily water)** | One sees a bird with a red eye, photographed from the back, swimming in oily water. | Swimming in oil is said to be good for the skin. Too bad, on the packaging no side effects were mentioned. | Swimming oil reduces swelling. As it appears to be a resting spot they lure seebirds. |
| **1560 (raptor)** | A raptor with black, red and white feathers flies downwards in an aggressive way. | Hugo is upset because the guests on his bird wedding don`t like his red and black suit. | In former times, falconlikes, accipitrids and owls were ranked as raptors. |
| **9571 (dead cat)** | A car is driving past a dead, wet cat lying on the side of the road. | We know it from our English lessons: sometimes it`s raining cats and dogs. | Carcasses should be removed immediately from the roadside as they are a source of danger for humans and animals. |
| **1121 (salamander)** | A salamander with red and white patterns and yellow eyes is sitting on a green leave. | Nasty: Nobody told Freddy that in case of fear of heights one should never look down. | Salamanders belong to the class of caudate amphibians without continuous fins. |
